# Prediction of SARS-CoV-2 Omicron Variant Immunogenicity, Immune Escape and Pathogenicity, through the Analysis of Spike Protein-Specific Core Unique Peptides

**DOI:** 10.3390/vaccines10030357

**Published:** 2022-02-24

**Authors:** Evangelos Kontopodis, Vasileios Pierros, Dimitrios J. Stravopodis, George T. Tsangaris

**Affiliations:** 1 Proteomics Research Unit, Biomedical Research Foundation of the Academy of Athens, 11527 Athens, Greece; kontopodisv@hotmail.gr (E.K.); pierrosv@gmail.com (V.P.); 2 Section of Cell Biology and Biophysics, Department of Biology, School of Science, National and Kapodistrian University of Athens, 15701 Athens, Greece; dstravop@biol.uoa.gr

**Keywords:** core unique peptide, COVID-19, immune escape, infectiveness, mutation, Omicron variant, pathogenicity, SARS-CoV-2, Spike protein, Uniquome

## Abstract

The recently discovered Omicron variant of the SARS-CoV-2 coronavirus has raised a new, global, awareness. In this study, we identified the Core Unique Peptides (CrUPs) that reside exclusively in the Omicron variant of Spike protein and are absent from the human proteome, creating a new dataset of peptides named as SARS-CoV-2 CrUPs against the human proteome (C/H-CrUPs), and we analyzed their locations in comparison to the Alpha and Delta variants. In Omicron, 115 C/H-CrUPs were generated and 119 C/H-CrUPs were lost, almost four times as many compared to the other two variants. At the Receptor Binding Motif (RBM), 8 mutations were detected, resulting in the construction of 28 novel C/H-CrUPs. Most importantly, in the Omicron variant, new C/H-CrUPs carrying two or three mutant amino acids were produced, as a consequence of the accumulation of multiple mutations in the RBM. These C/H-CrUPs could not be recognized in any other viral Spike variant. Our findings indicated that the virus binding to the ACE2 receptor is facilitated by the herein identified C/H-CrUPs in contact point mutations and Spike cleavage sites, while the immunoregulatory NF9 peptide is not detectably affected. Thus, the Omicron variant could escape immune-system attack, while the strong viral binding to the ACE2 receptor leads to the highly efficient fusion of the virus to the target cell. However, the intact NF9 peptide suggests that Omicron exhibits reduced pathogenicity compared to the Delta variant.

## 1. Introduction

The SARS-CoV-2 virus has a high mutagenesis frequency, hitherto producing 63 different variants with 39 considered as the most predominant forms, including Delta, the dominant variant of the 4th pandemic wave [1]. Recently, a new variant, Omicron (B.1.1.529), was identified in South Africa. Omicron is characterized by 30 amino acid changes, three small deletions, and one small insertion in Spike protein, as compared to the original virus, with 15 of them residing in the Receptor Binding Domain (RBD) from 319 to 541 amino acid residues [2].

In our previous studies, we have defined as Unique Peptides (UPs) the peptides whose amino acid sequence appears only in one protein across a given proteome. We also introduced the term of Core Unique Peptides (CrUPs), which are the peptides with a minimum amino acid sequence length that appear only in one protein across a given proteome, thus having a unique signature for a particular protein identification [3]. Therefore, each peptide of any size that contains a CrUP is considered a UP. Peptides of bigger sizes than CrUPs being constructed by continuous CrUPs are considered as Composite Core Unique Peptides (CmUPs). Hitherto, our results regarding the analysis of CrUPs in different species and organisms strongly suggest that CrUPs constitute a concrete group of peptides within a given proteome, with specialized properties and functions Thereby, we have introduced the new term “Uniquome”, which is defined as the total set of UPs belonging to a given proteome and serving as its unique molecular signature. Hence, to map the UP landscape of a proteome under examination, we have herein developed a novel and advanced bioinformatics tool, including big data analysis, and we have applied this tool for the analysis of Uniquome typifying all model organisms. In *Homo sapiens*, the analysis of the 20,430 reviewed proteins resulted in the identification of 7,263,888 CrUPs which construct the human Uniquome (hUniquome) ([3] and Kontopodis et al., 2022 (manuscript in preparation)).

Most importantly, to elucidate SARS-CoV-2 virus–host organism interactions, we have further designed a novel bioinformatics platform to analyze the Core Unique Peptides (CrUPs) of the SARS-CoV-2 virus against the human proteome (C/H-CrUPs) [1]. C/H-CrUPs represent a completely new set of peptides, which are the shortest in length peptides in a viral proteome that do not exist in the human proteome [3]. Based on their properties, the viral C/H-CrUPs could advance our knowledge regarding virus–host interactions, immune system response(s), and infectiveness and pathogenicity of the virus. Moreover, most importantly, they can be used as antigenic and diagnostic peptides, and likely druggable targets for successful therapeutic treatments.

In the present study, we have identified, cataloged, and analyzed Omicron-specific C/H-CrUPs in order to illuminate the mechanisms controlling infectivity, immune escape, and pathogenicity of the new variant.

## 2. Materials and Methods

### 2.1. Methods

In our previous, recent studies, we developed a bioinformatics tool that can extract the Core Unique Peptides (CrUPs) from a given proteome, thus creating its Uniquome (Figure S1) [1,3]. We have expanded this tool by introducing a new feature that can extract the CrUPs of each individual protein of a given proteome (target) versus the proteins of a reference proteome. This new feature, like the initial implementation, will split each protein in the target proteome to all possible peptides of length minimum (4 amino acids) to length maximum (100 amino acids), and search them against the reference proteome. Each search will exclude all peptides that contain a smaller peptide already identified as CrUP (Figure S2).

For the present study, we have engaged this new feature of our tool. We created a “custom” proteome consisting of sequences from all variants of the SARS-CoV-2 Spike proteins and used it as the target versus the human proteome. The tool produces as output the C/H-CrUPs per protein of the target proteome, thus revealing the CrUPs for each Spike variant versus the human proteome. 

Once we obtained the desired data, we ran a meta-analysis to identify how many C/H-CrUPs remained the same, or were added or lost on each variant versus the wild-type Spike protein. For this analysis, initially we took the identified C/H-CrUPs of the wild-type sequence and checked their presence against the respective C/H-CrUPs of the other variants. We only cared for the amino acid sequence and not the position this could be found within the protein. If the sequence was found, then we considered the peptide to be the same, otherwise we considered it to be lost on the examined variant. Next, we analyzed the identified C/H-CrUPs of each variant versus the wild-type sequence. If the peptide was detected only on the variant’s C/H-CrUPs, then we considered it as added. This meta-analysis also provided us with the position of each C/H-CrUP within the Spike protein, which we used to determine the area (e.g., RBD, RBM and S-cleavage site, as obtained by the Stanford COVID-19 Database) they resided in.

### 2.2. Databases

All proteomes and proteins were obtained from Uniprot. SARS-CoV-2 wild-type and variant sequences, and mutations were obtained from the Stanford COVID-19 Database (https://covdb.stanford.edu/page/mutation-viewer/, accessed on 23 December 2021).

## 3. Results and Discussion

### 3.1. Mapping the C/H-CrUPs Landscape of Spike Protein of the SARS-CoV-2 Omicron Variant 

SARS-CoV-2 virus seems to be highly mutated, so far producing more than 60 distinct variants. Hitherto, the highest pathogenic form is the Delta variant (B.1.617.2), with 10 different sub-variants. Recently, a novel variant called Omicron has been identified. It is characterized by 30 amino acid changes, three small deletions, and one small insertion in the Spike protein area, as compared to the wild-type viral respective sequence (Figure S3) [2]. Out of these genetic changes, 15 reside in the Receptor Binding Domain (RBD) from amino acid position 318 to 541, and two are located around the S-cleavage site(s) (Figure S3).

Advanced bioinformatics analysis of the Omicron variant Spike protein showed that it contains 983 C/H-CrUPs, a number that is comparable to the one of wild-type Spike proteins (987 C/H-CrUPs) and to the mean ± SD value of Spike protein-specific C/H-CrUPs (983 ± 2 C/H-CrUPs) (Table 1). Omicron variant Spike protein contains 34 mutations in total, which is the highest number of identified mutations among all virus variants.

These mutations seem to have a dramatic effect on the Spike protein C/H-CrUPs map. Compared to the wild-type Spike sequence, we found that 115 (new) C/H-CrUPs were created and 119 C/H-CrUPs were lost, almost twice as many when compared to the Alpha variant (51 and 56 C/H-CrUPs, respectively), and almost four times as many, compared to the other variants (Table 1). The distribution of these new C/H-CrUPs shows that the majority carry 6 amino acids in length (Figure S4).

### 3.2. Omicron-Specific C/H-CrUPs That belong to the Receptor Binding Domain

SARS-CoV-2 belongs to the β coronavirus group, which uses the plasma membrane receptor of Angiotensin-Converting Enzyme 2 (ACE2) to recognize and bind to the target cell [4]. The viral Spike protein attaches to ACE2 receptor by a Receptor Binding Domain (RBD) defined from amino acid position F318 to F541 [4,5]. The amino acid residues from W436 to Q506 inside RBD shape the Receptor Binding Motif (RBM), which carries 11 contact positions with ACE2 [5]. The RBD region has received great attention, as it seems to be a major target of antibodies against the virus and other therapeutic interventions [6,7,8]. 

**Figure 1:**
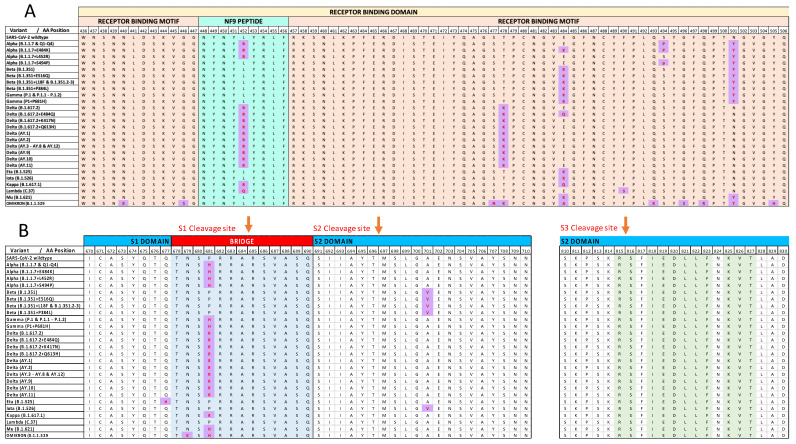
Mutations in different virus variants. (**A**) The mutations of the Receptor-Binding Motif (RBM) included in the Receptor-Binding Domain (RBD) are presented. (**B**) The mutations around the Spike cleavage sites are presented. Purple blocks mark the point mutation sites in the variants. Green colors indicate the Universal Peptides of the Spike proteins from Figure S2. Yellow colors mark the Receptor-Binding Domain of Spike protein interaction with ACE2. Pink colors mark the Receptor-Binding Motif. Cyan colors indicate the NF9 peptide, while light blue colors mark the Bridge between S1 and S2 domains. Red-colored arrows indicate the cleavage sites. With different colors in the upper side of the alignment, the different domains of the Spike protein are presented.

In the RBD region, the Omicron variant carries 15 mutations, 10 of which are identified in the RBM area (Figure 1A). This results in the identification of the highest number of newly constructed C/H-CrUPs in the RBD/RBM region, as compared to all other previous virus variants examined (Table S1). Table 2 describes all the new, herein identified, C/H-CrUPs of Omicron variant in Spike’s RBD region, in comparison to the Alpha and Delta variants, which represent two of the most predominant variants of the virus in human populations. Hence, it was proven that, in contrast to Alpha and Delta variants, at the end of Omicron variant RBM area from 440 to 508 amino acid position, 8 novel mutations were identified, resulting in the production of 28 new C/H-CrUPs. The most important finding is that in Omicron variant, for the first time, new C/H-CrUPs including two or three mutant amino acids were generated, with the peptides “QAGN*K*P”, “N*K*PCN”, “LK*SYS*F” and “K*SYS*FR*” being characteristic examples, as a result of the accumulation of multiple mutations in the positions 440, 446, 477, 478 and 493–505. These novel C/H-CrUPs that contain several mutated amino acids could not be found in any other virus variants previously. Taking into consideration recent data about virus infectivity, the multimutated, new, C/H-CrUP collection seems to radically change the structure and the epitope regions of end positions of the RBM area in the Omicron variant, causing a serious compromise of its antigenic capacity and facilitating the immune escape of the virus [9].

Remarkably, RBM area contains 11 out of the 12 contact points of viral Spike protein with the ACE2 cellular receptor. Among them, 7 contact points remained intact, while 4 mutations in positions Q493K, Q498R, N501Y and Y505H were identified, resulting in the construction of 17 new C/H-CrUPs (Table 3). N501Y mutation was found to be a major determinant of increased viral transmission, due to the improved binding affinity of Spike protein to ACE2 cellular receptor [10]. These findings indicate that virus binding to ACE2 receptor is notably affected by C/H-CrUP-specific mutations that can likely strengthen Spike-ACE2 protein–protein interaction(s).

Interestingly, an important amino acid sequence in the RBM area is the “*NYNYLYRLF*” peptide (from 448 to 456 position). This Tyrosine (Y)-enriched peptide carries two contact sites (Y449 and Y453), and it is known as the NF9 peptide [11]. It seems to affect antigen recognition, by being an immunodominant HLA*24:02-restricted epitope identified by CD8^+^ T cells. Of note, NF9 presents immune stimulation activity, and increases cytokine production derived from CD8^+^ T cells, such as IFN-γ, TNF-α and IL-2 [12]. In contrast to Delta, in the Omicron variant the NF9 amino acid content is not changed by any mutation detected, thus suggesting that the NF9 peptide could induce early immune system activation and efficient cytokine production, leading to a faster immune response, and thus reducing SARS-CoV-2 virus pathogenicity.

### 3.3. C/H-CrUPs Altered Architecture around the Spike-Cleavage Site(s) of the Omicron Variant

The molecular mechanism of Spike protein’s proteolytic activation has been shown to play a crucial role in the selection of host species, virus–cell fusion, and the viral infection of human lung cells [13,14,15]. Spike protein [SPIKE_SARS2 (P0DTC2)] contains three cleavage sites (known as S-cleavage sites) crucial for the virus fusion to the host cell: the R^685^↓S and R^815^↓S positions that serve as direct targets of the Furin protease, and the T^696^↓M position that can be recognized by the TMPRSS2 protease [16,17,18]. 

In these cleavage sites, the Omicron variant carries only the critical mutation P681H, which also appears in the Alpha variant (Figure 1B). Strikingly, in contrast to the Delta variant, which contains the P681R mutation, the P681H mutation constructs several new C/H-CrUPs in the Alpha and Omicron variants, thus indicating their dispensable contribution to virus fusion to the host cell (Table 4).

## 4. Conclusions

Core Unique Peptides constitute a distinct and important group of peptides within a proteome. The identification of CrUPs in an organism (e.g., virus, microbe, or mutant protein) against a distinct proteome of another organism is a completely novel approach, which could prove useful for the understanding of the action of microorganisms, the association of novel pharmacological targets with therapies, and the design of novel vaccines. It could be employed in many different kinds of diseases, such as cancer, athropozoans diseases, the design of vaccines for pathogenic viruses, and the identification of new antigenic epitopes capable for the development of new diagnostic or therapeutic antibodies. Therefore, we applied this dynamic and novel strategy, for the first time, in the identification of CrUPs derived from SARS-CoV-2 against the human proteome [1]. In that study, we analyzed all the CrUP peptides of all SARS-CoV-2 variants against the proteome of the host organism, which in our case was *Human sapiens*. Remarkably, this approach clearly revealed the immune escaping capacity, the contagious power and the high pathogenicity of Delta variant, in contrast to other variants. Notably, these findings have been confirmed by epidemiological data concerning the course of the disease.

In the present study, we engaged this approach to the analysis of the SARS-CoV-2 Omicron variant. The analysis of C/H-CrUP landscapes in the heavily mutated SARS-CoV-2 Omicron variant Spike protein unveiled that the Omicron variant, by the generation of novel multi-mutated C/H-CrUPs, could escape the immune system defense mechanisms, while these C/H-CrUP-specific mutations could facilitate more efficient virus binding to the ACE2 cellular receptor, and a more productive fusion of the virus to the host cell. Most importantly, in contrast to the Delta variant, the intact NF9 peptide in the Omicron variant, which has a known immunostimulatory effect, suggests that Omicron exhibits reduced pathogenicity as compared to Delta.

## Figures and Tables

**Table 1:** SARS-CoV-2 Spike protein C/H-CrUPs across variants, as compared to the wild-type virus respective sequence.

	Spike Protein						
Variant	C/H-CrUPs	Same C/H-CrUPs	% of Same C/H-CrUPs	New C/H-CrUPs	% of New C/H-CrUPs	Lost C/H-CrUPs	% of Lost C/H-CrUPs
**Wild-type virus**	987						
**Alpha (B.1.1.7) + (Q1-Q4)**	982	931	94.8	51	5.2	56	5.7
**Alpha (B.1.1.7 + E484K)**	983	928	94.4	55	5.6	59	6.0
**Alpha (B.1.1.7 + L452R)**	981	936	95.4	45	4.6	51	5.2
**Alpha (B.1.1.7 + S494P)**	981	936	95.4	45	4.6	51	5.2
**Beta (B.1.351)**	981	954	97.2	27	2.8	33	3.3
**Beta (B.1.351 + E516Q)**	981	949	96.7	32	3.3	38	3.8
**Beta (B.1.351 + L18F) (B.1.351.2-3)**	979	948	96.8	31	3.2	39	3.9
**Beta (B.1.351 + P384L)**	980	949	96.8	31	3.2	38	3.9
**Gamma (P.1) (P.1.1 - P.1.2)**	985	930	94.4	55	5.6	57	5.8
**Gamma (P1 + P681H)**	985	930	94.4	55	5.6	57	5.8
**Delta (B.1.617.2)**	984	948	96.3	36	3.7	39	4.0
**Delta (B.1.617.2 + E484Q)**	984	945	96.0	39	3.4	42	4.3
**Delta (B.1.617.2 + K417N)**	984	944	95.9	40	4.1	43	4.4
**Delta (B.1.617.2 + Q613H)**	984	947	96.2	37	3.8	40	4.1
**Delta (AY.1)**	984	944	95.9	40	4.7	43	4.1
**Delta (AY.2)**	985	939	95.3	46	4.8	48	4.9
**Delta (AY.3 - AY.8) + (AY.12)**	983	951	96.7	32	3.3	36	3.7
**Delta (AY.9)**	983	951	96.7	32	3.3	36	3.6
**Delta (AY.10)**	983	951	96.7	32	3.3	36	3.6
**Delta (AY.11)**	983	951	96.7	32	3.3	36	3.6
**Eta (B.1.525)**	990	956	96.5	34	3.4	31	3.1
**Iota (B.1.526)**	984	960	97.5	24	2.4	27	2.7
**Kappa (B.1.617.1)**	985	964	97.8	21	2.1	23	2.3
**Lambda (C.37)**	982	949	96.6	33	3.4	38	3.9
**Mu (B.1.621)**	983	953	96.9	30	3.1	34	3.4
**Omicron (B.1.1.529)**	983	868	88.3	115	11.7	119	12.1

**New C/H-CrUPs** is the number of new constructed peptides of each variant compared to C/H-CrUPs of wild-type virus; **% of new C/H-CrUPs** is the % of new constructed peptides compared to the total C/H-CrUPs number of each variant; **Lost C/H-CrUPs** is the number of peptides lost in each variant compared to C/H-CrUPs of wild-type virus; **% of lost C/H-CrUPS** is the % of lost peptides compared to the total C/H-CrUPs number of each variant.

**Table 2:** C/H-CrUPs constructed aroud the mutations in RBD of Alpha, Delta and Omicron SARS-CoV-2 variants.

**Alpha Variant**						**Delta Variant**				
**C/H-CrUP**	**Position**	**Mutation**	**New C/H-CrUPs**	**Position**		**C/H-CrUP**	**Position**	**Mutation**	**New C/H-CrUPs**	**Position**
GNYNYL	447	L452R	GNYNYR	447		PGQTGKI	412	K417N		
NYNYLY	448					GQTGKIA	413		GQTGNI	413
			YNYRY	449					QTGNIA	414
NYLYRL	450		NYRYRL	450		TGKIAD	415		TGNIAD	415
YLYRLF	451		YRYRLF	451		GKIADY	416		GNIADY	416
LYRLFR	452									
						GNYNYL	447	L452R	GNYNYR	447
CNGVEG	480	E484K	CNGVKG	480		NYNYLY	448			
NGVEGF	481		NGVKGF	481					YNYRY	449
GVEGFN	482		GVKGFN	482		NYLYRL	450		NYRYRL	450
			KGFNC	484		YLYRLF	451		YRYRLF	451
						LYRLFR	452			
YFPLQS	489	S494P N501Y	YFPLQP	489						
FPLQSY	490		FPLQPY	490		YQAGST	473	T478K	YQAGSK	473
PLQSYG	491		PLQPYG	491					QAGSKP	474
QSYGF	493		QPYGF	493		AGSTPC	475		AGSKPC	475
SYGFQP	494		PYGFQP	494					GSKPCN	476
GFQPTN	496					STPCN	477			
FQPTNG	497								KPCNG	478
QPTNGV	498									
			QPTΥ	498						
PTNGVG	499		PTΥG	499						
TNGVGY	500		TΥGV	500						
NGVGYQ	501		ΥGVG	501						
**Omicron variant**										
**C/H-CrUP**	**Position**	**Mutation**	**New C/H-CrUPs**	**Position**		**C/H-CrUP**	**Position**	**Mutation**	**New C/H-CrUPs**	**Position**
NLCPFG	334	G339D	NLCPFD	334		IYQAGS	472	S477N T478K		
LCPFGE	335		LCPFDE	335		YQAGST	473		YQAGN	473
PFGEVF	337		PFDEV	337					QAGNKP	474
FGEVFN	338		FDEVFN	338		AGSTPC	475			
GEVFNA	339		DEVFNA	339		STPCN	477		NKPCN	477
									KPCNG	478
VLYNSA	367	S371L S373P S375F	VLYNLAP	367						
LYNSAS	368					CNGVEG	480	E484A	CNGVAG	480
YNSASF	369		YNLAPF	369		NGVEGF	481		NGVAGF	481
NSASFST	370					GVEGFN	482		GVAGFN	482
			LAPFFT	371					VAGFNC	483
ASFSTF	372		APFFTF	372						
SFSTFK	373					CYFPLQ	488	Q493K G496S Q498R N501Y Y505H	CYFPLK	488
			FFTFK	374		YFPLQS	489		YFPLKS	489
STFKC	375		FTFKCY	375		FPLQSY	490		FPLKSY	490
						PLQSYG	491			
PGQTGKI	412	K417N							LKSYSF	492
GQTGKIA	413		GQTGNI	413		QSYGF	493		KSYSFR	493
			QTGNIA	414		SYGFQP	494		SYSFRP	494
TGKIAD	415		TGNIAD	415		YGFQPT	495		YSFRPT	495
GKIADY	416		GNIADY	416		GFQPTN	496			
						FQPTNG	497		FRPTY	497
WNSNN	436	N440K G446S	WNSNKL	436		QPTNGV	498		RPTYGV	498
SNNLDS	438		SNKLDS	438		PTNGVG	499			
NNLDSK	439		NKLDSKV	439		TNGVGY	500		TYGVGH	500
			KLDSKVS	440		NGVGYQ	501			
LDSKVG	441					GVGYQP	502		GVGHQ	502
DSKVGG	442		DSKVSG	442		VGYQPY	503		VGHQPY	503
KVGGNY	444		KVSGNY	444		GYQPYR	504			
VGGNYN	445		VSGNYN	445		YQPYRV	505		HQPYR	505
GGNYNY	446									

The original and newly constructed C/H-CrUPs around the native and mutant sites of RBD region of SARS-CoV-2 Spike protein in Alpha, Delta and Omicron variants are presented. With the red colors, the mutant amino acids in wild-type C/H-CrUPs and in the newly constructed peptides are marked.

**Table 3:** C/H-CrUPs around SARS-CoV-2 RBD contact positions.

	WILD-TYPE							OMICRON VARIANT				
Contact Positions	C/H-CrUPs						Mutations	Newly Constructed C/H-CrUPs				
N439	AWNSN	WNSNN	SNNLDS	NNLDSK								
Y449	KVGGNY	VGGNYN	GGNYNY	GNYNYL	NYNYLY							
Y453	NYNYLY	NYLYRL	YLYRLF	LYRLFR	YRLFRK							
F486	NGVEGF	GVEGFN	GFNCY	FNCYF								
N487	GVEGFN	GFNCY	FNCYF									
Y489	GFNCY	FNCYF	CYFPLQ	YFPLQS								
Q493	CYFPLQ	YFPLQS	FPLQSY	PLQSYG	QSYGF		Q493K	CYFPLK	YFPLKS	FPLKSY	LKSYSF	KSYSFR
Q498	SYGFQP	YGFQPT	GFQPTN	FQPTNG	QPTNGV		Q498R	KSYSFR	SYSFRP	YSFRPT	FRPTY	RPTYGV
T500	YGFQPT	GFQPTN	FQPTNG	QPTNGV	PTNGVG	TNGVGY						
N501	GFQPTN	FQPTNG	QPTNGV	PTNGVG	TNGVGY	NGVGYQ	N501Y	FRPTY	RPTYGV	TYGVGH		
Y505	TNGVGY	NGVGYQ	GVGYQP	VGYQPY	GYQPYR	YQPYRV	Y505H	TYGVGH	GVGHQ	VGHQPY	HQPYR	

The original and newly constructed C/H-CrUPs residing around the native and contact positions of the SARS-CoV-2 Spike protein RBD region. The C/H-CrUPs of wild-type and Omicron variant are presented. With red colors, the mutant amino acids in wild-type C/H-CrUPs and in the newly constructed peptides are marked.

**Table 4:** C/H-CrUPs arround the Spike protein cleavage sites.

Cleavage Site	Mutation	Variant	Position	New C/H-CrUPs
R^685^↓S	P681R	Delta	680	SRRRAR↓S
	P681H	Alpha Omicron	677	QTNSH
			678	TNSHR
			680	SHRRAR
T^696^↓M	A701V	Beta	None	
R^815^↓S	None	None	None	

C/H-CrUPs around the mutant positions of Spike protein cleavage sites are presented. Symbol “↓” indicates the protein cleavage positions.

## Data Availability

All data of the present article are available in the main text or in the supplementary materials.

## Supplementary Materials

The following supporting information can be downloaded at: https://www.mdpi.com/article/10.3390/vaccines10030357/s1. Figure S1: Uniquome creation algorithm; Figure S2: Extracting CrUPs of Target vs Reference Proteome; Figure S3: Alignment of the SARS-CoV-2 Spike protein (SPIKE_SARS2, P0DTC2) of the 26 variants, together with the wild-type Spike Protein (SPIKE_SARS2, P0DTC2); Figure S4: Length distribution of Omicron variant Spike protein C/H-CrUPs; Table S1: New C/H-CrUPs located in the RBD and RBM regions of the Spike protein across virus variants.

## Abbreviations

UPs: Unique Peptides; CrUPs: Core Unique Peptides; C/H-CrUPs: SARS-CoV-2 Core Unique Peptides against Human Proteome; RBD: Receptor Binding Domain; RBM: Receptor Binding Motif.
