# Supplementary material for: Prediction of SARS-CoV-2 Omicron Variant Immunogenicity, Immune Escape and Pathogenicity, through the Analysis of Spike Protein-Specific Core Unique Peptides
Source: Vaccines (Basel). 2022 Feb 24;10(3):357. doi: 10.3390/vaccines10030357 (PMC8955659; doi:10.3390/vaccines10030357)
Supplement: Supplementary file 1 [file vaccines-10-00357-s001.zip › vaccines-1586875-supplementary.pdf]

vaccines

MDPI

SUPPLEMENTARY DATA

Figure S1: Uniquome creation algorithm, **Figure S2:** Extracting CrUPs of Target vs Reference Proteome, **Figure S3:** Alignment of the SARS-CoV-2 spike protein (SPIKE_SARS2, P0DTC2) of the 26 variants, together with the wild-type Spike Protein (SPIKE_SARS2, P0DTC2), **Figure S4:** Length distribution of Omicron variant Spike protein C/H-CrUPs, **Table S1**: New C/H-CrUPs in the RBD and RBM regions of the Spike protein across virus variants.

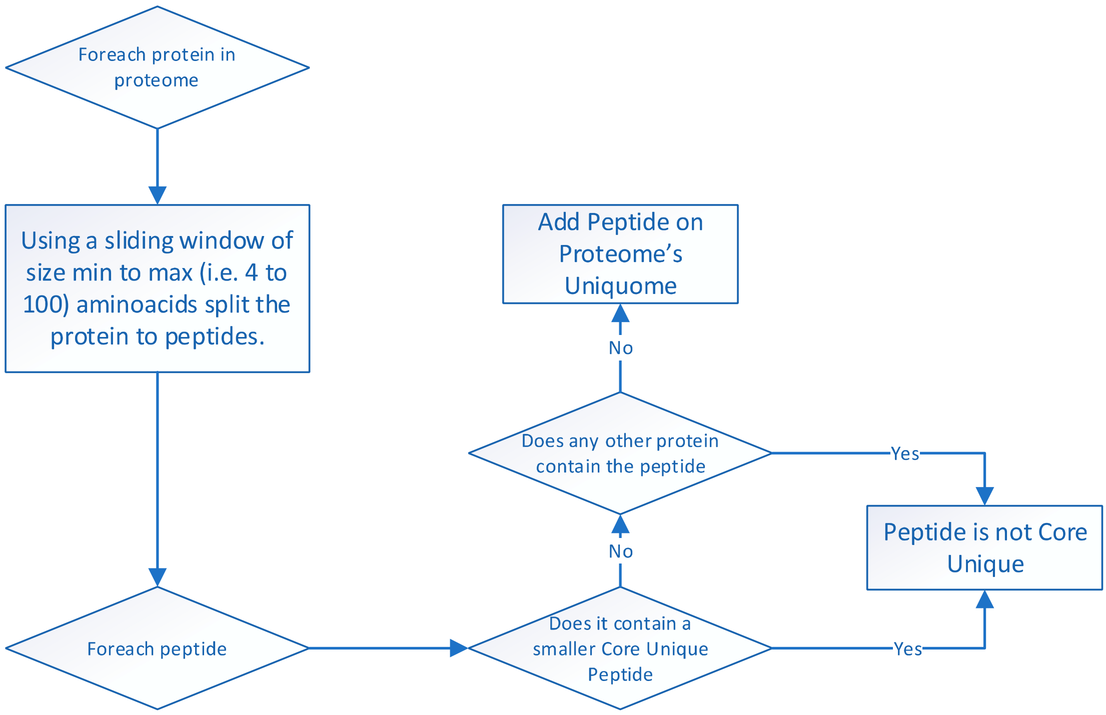

Figure S1: Uniquome creation algorithm.

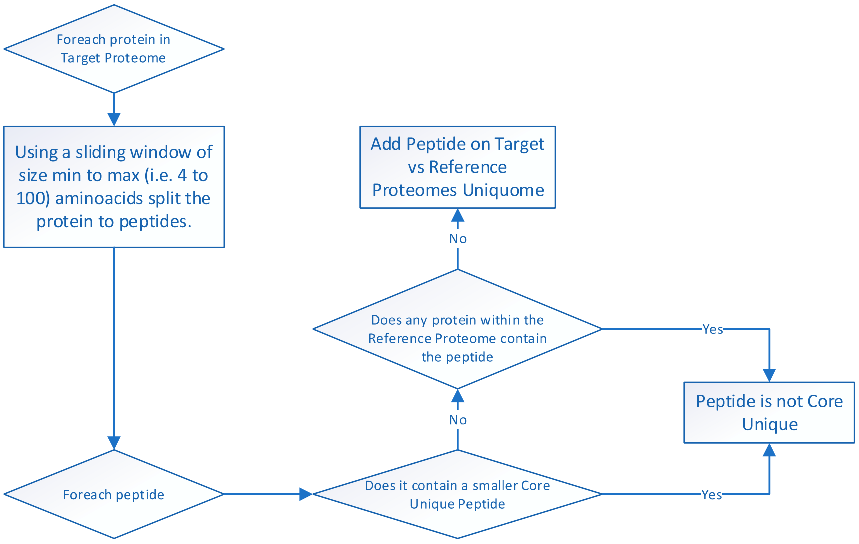

Figure S2: Extracting CrUPs of Target vs Reference Proteome.

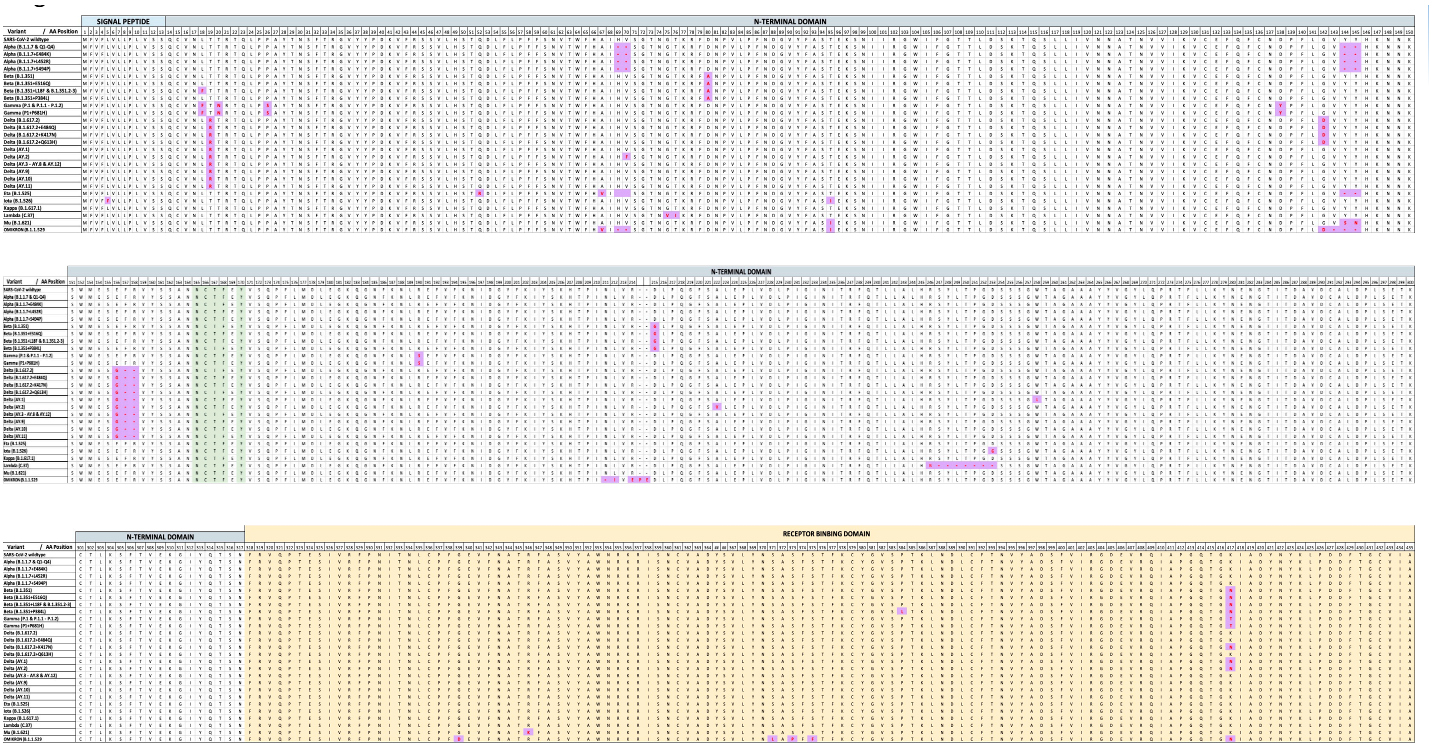

Figure S3. Cont.

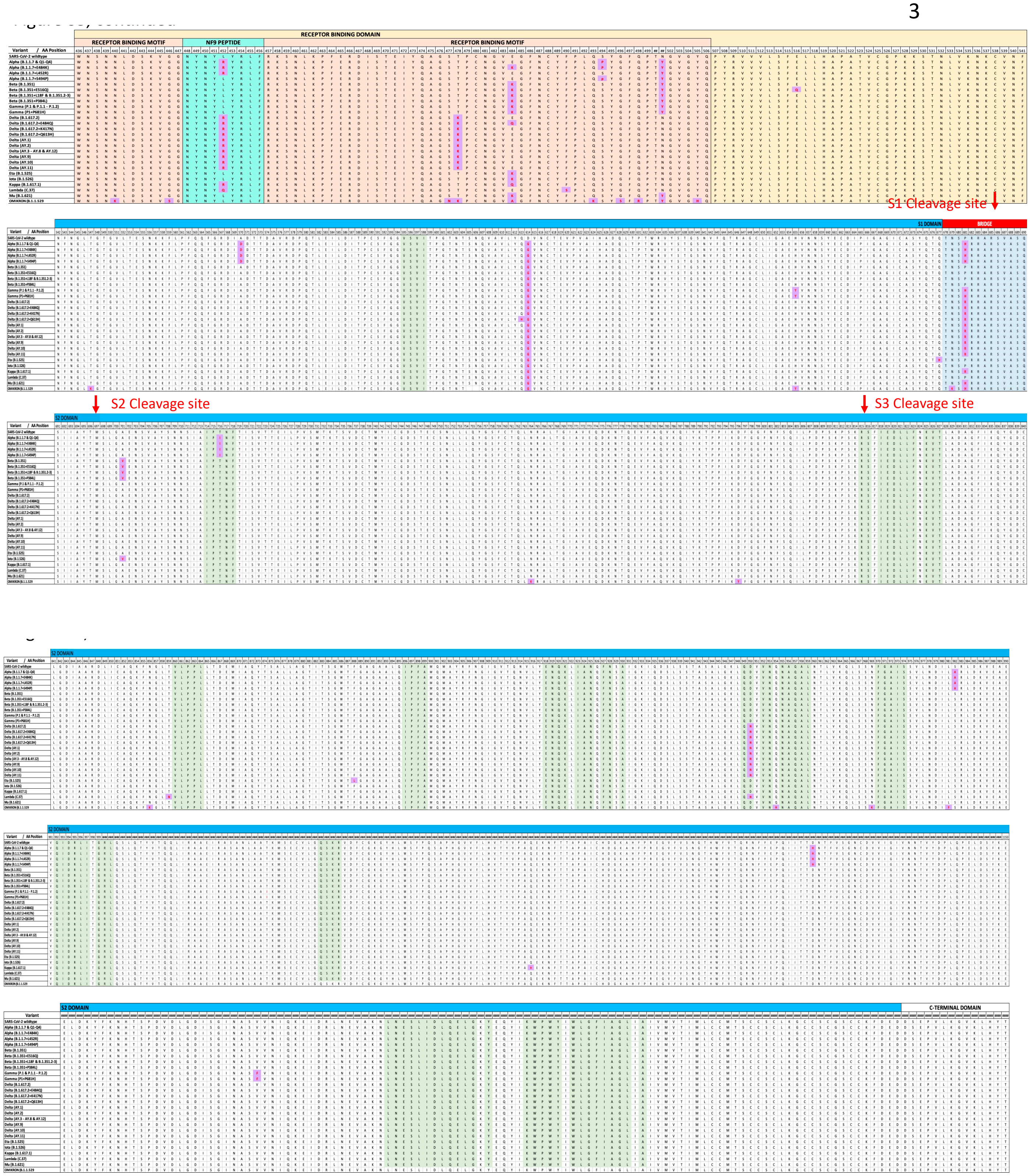

Figure S3. Alignment of the SARS-CoV-2 spike protein (SPIKE_SARS2, P0DTC2) of the 26 variants, together with the wild-type Spike Protein (SPIKE_SARS2, P0DTC2). The complete spike sequence alignment. Purple blocks marked the point mutations sites in the variants, green color indicate the Universal Peptides of the spike proteins from Fig. S2. Yellow color mark the Receptor-Binding Domain of spike protein to ACE2, pink color mark the Receptor-Binding Motif, cyan mark the NF9 peptide and light blue mark the Bridge between S1 and S2 domain. Red arrows

indicate the cleavage sites. With different colors in the upper side of the alignment, the different domains of the spike protein are marked.

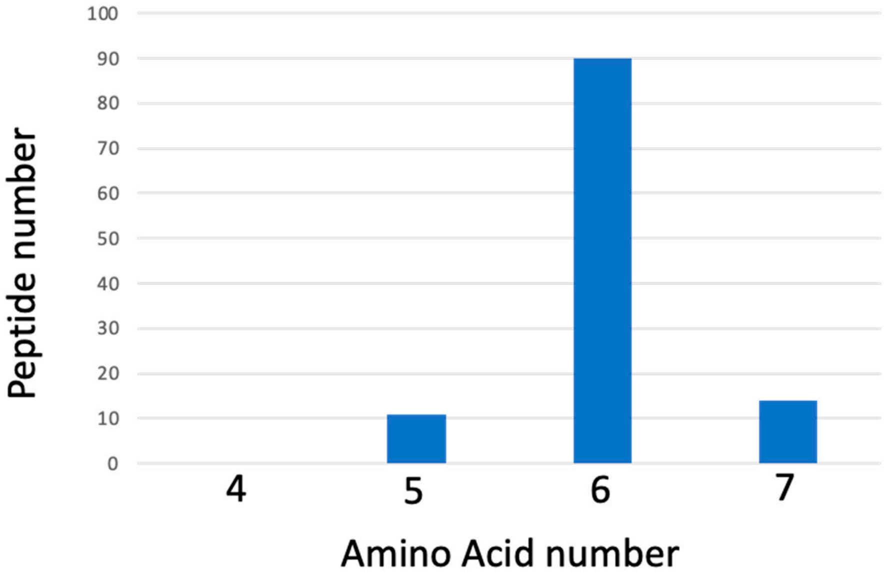

Figure S4. Length distribution of Omicron variant Spike protein C/H-CrUPs.

Table S1: New C/H-CrUPs in the RBD and RBM regions of the Spike protein across virus variants.

Variant	New C/H-CrUPs in Spike protien	New C/H-CrUPS in the RBD region	% of new C/H CrUPs in the RBD region	New C/H-CrUPS in the RBM region	% of new C/H CrUPs in the RBM region
Alpha (B.1.1.7) + (Q1-Q4)	51	13	25%	13	25%
Alpha (B.1.1.7+E484K)	55	17	31%	17	31%
Alpha (B.1.1.7+L452R)	45	7	16%	7	16%
Alpha (B.1.1.7+S494P)	45	7	16%	7	16%
Beta (B.1.351)	27	11	41%	7	26%
Beta (B.1.351+E516Q)	32	16	50%	7	22%
Beta (B.1.351+L18F) (B.1.351.2-3)	31	11	35%	7	23%
Beta (B.1.351+P384L)	31	15	48%	7	23%
Gamma (P.1) (P.1.1 - P.1.2)	55	11	20%	7	13%
Gamma (P1+P681H)	55	11	20%	7	13%
Delta (B.1.617.2)	36	9	25%	9	25%
Delta (B.1.617.2+E484Q)	39	12	31%	12	31%
Delta (B.1.617.2+K417N)	40	13	33%	9	23%
Delta (B.1.617.2+Q613H)	37	9	24%	9	24%
Delta (AY.1)	40	13	33%	9	23%
Delta (AY.2)	46	13	28%	9	20%
Delta (AY.3 - AY.8) + (AY.12)	32	9	28%	9	28%
Delta (AY.9)	32	9	28%	9	28%
Delta (AY.10)	32	9	28%	9	28%
Delta (AY.11)	32	9	28%	9	28%
Eta (B.1.525)	34	4	12%	4	12%
Iota (B.1.526)	24	4	17%	4	17%
Kappa (B..1.617.1)	21	7	33%	7	33%
Lambda (C.37)	33	9	27%	9	27%
Mu (B.1.621)	30	12	40%	7	23%
Omicron (B.1.1.529)	115	47	41%	27	23%
